# Amine-crosslinked lignin for water pollution attributable to organic dye remediation: Versatile adsorbent for selective dye removal and reusability

**DOI:** 10.1016/j.heliyon.2024.e37497

**Published:** 2024-09-05

**Authors:** Do Hun Oh, Ji Won Heo, Qian Xia, Min Soo Kim, Yong Sik Kim

**Affiliations:** Department of Paper Science & Engineering, College of Forest and Environmental Sciences, Kangwon National University, Chuncheon, 24341, Republic of Korea

**Keywords:** Kraft lignin, Amination, Adsorption mechanism, Organic dye remediation

## Abstract

Lignin, an abundant natural resource, has not been effectively utilized. In this study, the functionality of lignin was enhanced through amination to produce amine-crosslinked lignin, and its adsorption behavior toward cationic and anionic dyes was investigated. Chemical structure analysis confirmed the successful introduction of amine groups, thereby improving the molecular weight and thermal stability of the optimized amine-crosslinked lignin. Additionally, the amine-crosslinked lignin exhibited a larger specific surface area than kraft lignin, as well as excellent adsorption capacity for both anionic and cationic dyes. Furthermore, it selectively adsorbed anionic and cationic dyes depending on pH conditions. The adsorption kinetics were described using a pseudo-second-order model, and the adsorption isotherms for congo red and methyl green were determined using the Langmuir and Freundlich equations, respectively. Additionally, the reusability and adsorption efficiency of the optimized amine-crosslinked lignin were evaluated, confirming its stable and repeatable adsorption efficiency for congo red and methyl green even after five repeated cycles. The assumed adsorption mechanism was attributed to electrostatic interactions. Therefore, the successful synthesis and excellent adsorption properties of amine-crosslinked lignin suggest its promising potential for environmentally friendly and efficient removal of both cationic and anionic dyes, thereby offering a sustainable solution for wastewater treatment and remediation.

## Highlights

• Amine-crosslinked lignins were successfully prepared.
• Amine-crosslinked lignins had different surface charges under different pH values.
• Amine-crosslinked lignins can selectively adsorb cationic/anionic dyes.
• The q_max_ for CR and MG were 280.77 and 545.95 mg·g^−1^, respectively.

## Graphical abstract

**Figure:**
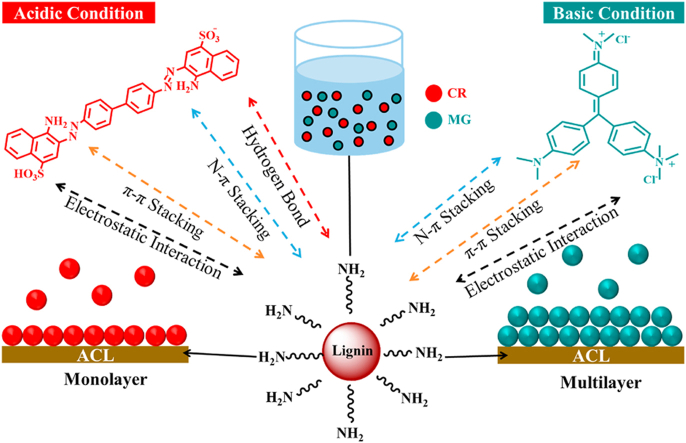

## 1 Introduction

The rapid growth of modern industries has increased the production and use of chemical substances, including organic dyes, resulting in water pollution becoming a global issue [1]. Organic dyes from various industries, such as the textile, paper, and plastic industries, are significant contributors to water pollution [2]. Annually, 10–15 % of the 700,000 tons of organic dyes are discharged into wastewater without proper treatment, posing a serious threat to aquatic ecosystems [3]. These dyes deplete the dissolved oxygen in the aquatic environment, causing imbalance and hindering sunlight penetration, thereby disrupting the photosynthetic activities and negatively affecting flora and fauna [4]. Furthermore, these organic dyes can transform into toxic substances with carcinogenic effects during metabolic processes [5].

Research has been actively conducted to efficiently and economically remove organic dyes from wastewater [6]. Wastewater treatment involves various physical, chemical, and biological processes. Adsorption, coagulation, electrochemical processes, and oxidation are the most commonly used methods for treating dye wastewater [7]. Among these, adsorption has received considerable attention owing to its simplicity and rapid application in various forms [8]. However, activated carbon, which is an existing adsorbent, is limited by its cost and difficulty in reusing. In addition, its efficiency in specific environments is limited [9]. Therefore, developing efficient and economical biomass adsorbents is necessary.

Lignin is a complex compound that is a major constituent of plant cell walls in wood and other plant fibers [10]. It possesses a three-dimensional network structure comprising phenolic, hydroxyl, carboxyl, benzyl alcohol, methoxyl, and aldehyde groups, endowing it with excellent potential as an adsorbent [11]. Moreover, it has a large surface area and can effectively adsorb various compounds owing to its diverse binding capabilities [11]. As such, lignin has been gaining attention as an environmentally friendly solution with high biodegradability, economic viability, and recyclability [12].

The introduction of functional groups to lignin further enhances its functionality. Zhang et al. (2016b) achieved a maximum adsorption capacity of 20.38 mg·g^−1^ for methylene blue removal using high-quality lignin extracted from steam-exploded straw containing high phenolic and aliphatic hydroxyl contents [13]. Among these functional groups, amino groups can adsorb both cationic and anionic species in aqueous solutions, thereby enhancing the adsorption capacity of lignin and overcoming its drawbacks [14]. Chen et al. employed the amination of alkaline lignin via the Mannich reaction, producing aminated lignin aerogel with a maximum adsorption capacity of 113.5 mg·g^−1^ for malachite green dye [15]. An et al. synthesized silanized and aminated lignin and achieved a maximum adsorption capacity of 74.4 mg·g^−1^ for congo red (CR) dye [14].

In this study, we enhanced the functionality of lignin as an adsorbent by introducing amino groups to 2-chloroethylamine hydrochloride (CEH), thereby obtaining amine-crosslinked lignin (ACL). Subsequently, we characterized ACL using elemental analysis, Fourier-transform infrared (FT-IR) spectroscopy, gel permeation chromatography (GPC), X-ray photoelectron spectroscopy (XPS), thermogravimetric analysis (TGA), scanning electron microscopy (SEM), energy-dispersive X-ray spectrometry (EDS), zeta-potential measurements, and particle-size analysis. CR and methylene green (MG) dyes were used in this study owing to their contrasting ionic properties; that is, CR is an anionic dye, and MG is a cationic dye. This choice enabled the evaluation of the effectiveness of the adsorbent across a wide range of dye molecules. Additionally, both dyes are commonly employed in industrial applications; however, they exhibit toxicity, potential carcinogenic effects, and poor biodegradability, thereby contributing to environmental pollution. The effects of pH, time, temperature, and concentration on the adsorption were investigated. Furthermore, the relationship between adsorption capacity and mechanism was studied using the Langmuir and Freundlich isotherm models.

## 2 Materials and methods

### 2.1 Materials

Kraft lignin (KL), produced from the kraft pulping of softwood chips, was provided by Tiger Forest & Paper Group Co., Ltd. (China). Black liquor was acidified to precipitate lignin, which was then purified by washing with water. Finally, the lignin was dewatered using a filter press and dried naturally. CEH (99 %) and CR were purchased from Sigma-Aldrich (USA). Ethanol (>94.5 %), HCl (36 %), and NaOH (98 %) were purchased from Dae-Jung Chemical & Metals Co., Ltd. (South Korea). MG was purchased from Tokyo Chemical Industry Co., Ltd. (Tokyo, Japan).

### 2.2 Amination of lignin

The amination of KL by CEH was performed in a 250 mL round-bottom flask equipped with a reflux condenser and magnetic stirrer (Reaction 1). Specifically, KL (3 g) was dissolved in 2 M NaOH solution (100 mL). The reaction was initially performed at various reaction times to compare the effects of time, whereby the optimal reaction time was selected. Subsequently, 0.041, 0.055, and 0.111 mol CEH were added to the reaction mixture. The mixture was heated to 80 °C and stirred for 24 h to induce ACL substitution. After the reaction, the pH of the mixture was adjusted to 7.0 using 2 mol·L^−1^ HCl. The resulting mixture was transferred to a cellulose separation bag (Cellu-Sep H1, molecular weight cutoff: 1,000, USA) and immersed in excess deionized water in a container. Deionized water was periodically replaced, and the separation bag was stirred continuously for 72 h. Subsequently, the solution was filtered, and the resulting filtrate was freeze-dried to obtain ACL.

### 2.3 Characterization of lignin samples

The chemical structures of the lignin samples were analyzed by FT-IR spectroscopy (Frontier, PerkinElmer, USA) in the attenuated total-reflectance mode. The spectra were recorded in the range of 500–4000 cm^−1^ at a resolution of 4.0 cm^−1^ with 64 scans [16]. Proton nuclear magnetic resonance (^1^H NMR) spectra were recorded on a 600-MHz Fourier-transform nuclear magnetic resonance (FT-NMR) spectrometer (Bruker Avance Neo 600, Germany). ^1^H NMR spectra were recorded by dissolving the lignin samples (20 mg) in 1 % NaOH/D_2_O. Additionally, acetylated lignin samples (20 mg) were dissolved in dimethyl sulfoxide-*d_6_* to record ^1^H NMR spectra [17]. The molecular weights were measured via GPC (Shimadzu-20A, Japan) equipped with PLgel columns (PLgel 5-μm MIXED-C and -D, and PLgel 3-μm MIXED-E, Agilent Technologies, USA) and an ultraviolet (UV) detector with N, N-dimethylformamide containing 0.1 % LiBr as the eluent. An elemental analyzer (Thermo Scientific, FlashEA1112, UK) equipped with a thermal conductivity detector was used to measure the contents of different elements (C, H, and N) in the lignin samples [17]. The chemical states of the elements on the surfaces of the lignin samples were analyzed using XPS (K-Alpha+, Thermo Scientific, UK) [18,19]. The N_2_ adsorption–desorption experiments (QUADRASORB-SI-KR/MP, Quantachrome Instruments, USA) were performed at 77.3 K to calculate the specific surface areas of the lignin samples using the Brunauer–Emmett–Teller method [20]. The pore volumes of the lignin samples were determined from the amount of adsorbed N_2_ at a relative pressure of 0.99. The pore-size distribution of the lignin samples was determined from the adsorption isotherm data using the Barrett–Joyner–Halenda (BJH) method [21]. The zeta potentials (Zetasizer Nano S, Malvern Instruments, UK) of the lignin samples were measured in the pH range of 3–10 by preparing different suspensions of the lignin samples (0.025 %, w/w) in a 0.001 M NaCl solution with the pH adjusted using 0.1 M HCl and NaOH solutions [22]. The morphologies and elemental compositions of the lignin sample surfaces were determined by SEM coupled with EDS. The lignin samples for FT-IR, NMR, and GPC analyses were prepared by acetylation following a previously reported method [23]. Lignin samples (100 mg) were dissolved in a mixture of pyridine (2 mL) and acetic anhydride (2 mL), and the solution was stirred at 25 °C for 24 h. After the reaction, the solution was poured into ice-cold water (100 mL) under continuous stirring. The precipitated lignin was filtered through a nylon membrane (0.20 μm, 47 mm) and washed with deionized water. The isolated lignin was washed again with deionized water and finally dried at 60 °C.

### 2.4 Dye adsorption experiments

Various factors, including the pH of the dye solution, adsorption time, initial dye concentration, and temperature, were investigated in the dye adsorption experiments. ACL (30 mg) and dye solution (30 mL) were mixed. Subsequently, dye adsorption was performed at various pH values (3–10) in a shaking incubator (IST-4075, JEIO Tech, Korea). After adsorption, the supernatant was collected via centrifugation. The dye concentration in the supernatant was determined using UV spectrophotometry (UV-2550, Shimadzu, Japan). The absorbance values were recorded at the maximum absorption wavelengths of CR (497 nm) and MG (633 nm). The adsorption capacity (Q) and efficiency (E) for CR and MG adsorptions were calculated using Equations (1), (2), respectively [24].:

(1) $$Q\;\left(\text{mg}\cdot g^{-1}\right)=\left(C_{i}\;-\;C_{f}\right)\times V/M\text{,}$$

(2) $$E\;\left(\%\right)=\left[\left(C_{i}\;-\;C_{f}\right)\;/\;C_{i}\right]\times 100\text{,}$$

where C_*i*_ and C_*f*_ are the initial and final dye concentrations in the solution (mg·L^−1^), respectively; *V* is the volume of the dye solution (L); and *M* is the weight of the adsorbent (g).

### 2.5 Reusability

A series of adsorption/desorption cycles for CR and MG were conducted five times to assess the reusability of ACL. Each cycle consisted of an adsorption test, followed by washing (desorption process) with a distilled water (DW)/ethanol mixture in a 5:5 ratio. After the adsorption test, the ACL-2 content was filtered, soaked in DW/ethanol (500 mL) for 15 min, and washed by filtration and vacuum pumping. This process was repeated five times to achieve desorption, and the remaining ACL-2 sample on the filter paper was dried in an oven at 70 °C for 18 h. The entire procedure was repeated four more times, and the removal efficiency was compared with the initial value.

## 3 Results and discussion

### 3.1 Characteristics of ACL

#### 3.1.1 Effect of CEH dosage on the nitrogen content, and molecular weight of ACL

**Scheme 1:**
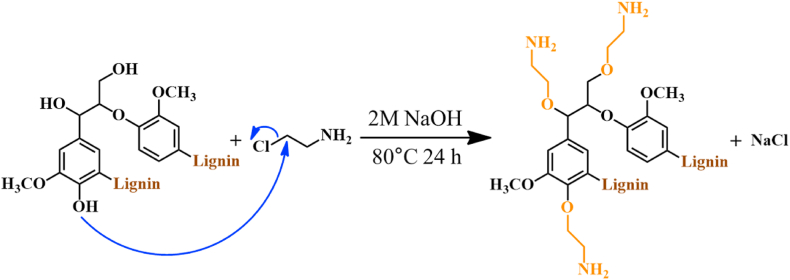
Synthesis of ACL.

KL was aminated using CEH to synthesize ACL-containing amino groups (Scheme 1). The elemental composition of ACL, which varied with time, is summarized in Table S1. The nitrogen content increased with time, reaching a maximum value of 3.6 % after 24 h, which was selected as the fixed reaction time for the subsequent experiments. With the fixed reaction time of 24 h, the elemental composition and molecular weight of ACL, which varied with the CEH dosage, are summarized in Table 1. An increase in the CEH dosage increased the nitrogen content of the ACL. The molecular weights of KL and ACL were measured by GPC. Prior to measurement, the lignin samples were acetylated to enhance their solubility in organic solvents [25]. The number-average molecular weight (Mn) and weight-average molecular weight (Mw) of ACL-1 and ACL-2 are significantly higher than those of KL. Particularly, the Mw values are substantially different, which can be attributed to the reaction of NaOH with CEH to form aziridine, thereby inducing the crosslinking of giant lignin molecules [26]. Molecular-weight measurements were not performed for ACL-3 because of solubility problems owing to cross-linking.

**Table 1:** Elemental contents and molecular weights of the KL and ACL samples.

Entry	CEH (mol)	Elemental content			Molecular weight		
		C (%)	N (%)	S (%)	M_n_ (g·mol^−1^)	M_w_ (g·mol^−1^)	PDI
KL	0	66.32	0.4	1.89	9200	16700	1.82
ACL-1	0.041	63.46	2.3	1.54	13800	63600	4.61
ACL-2	0.055	63.10	3.6	0.76	14900	52000	3.49
ACL-3	0.111	58.20	6.1	1.43	NA*	NA*	NA*

NA* denotes “not available.”

#### 3.1.2 Chemical structure of ACL

**Fig. 1:**
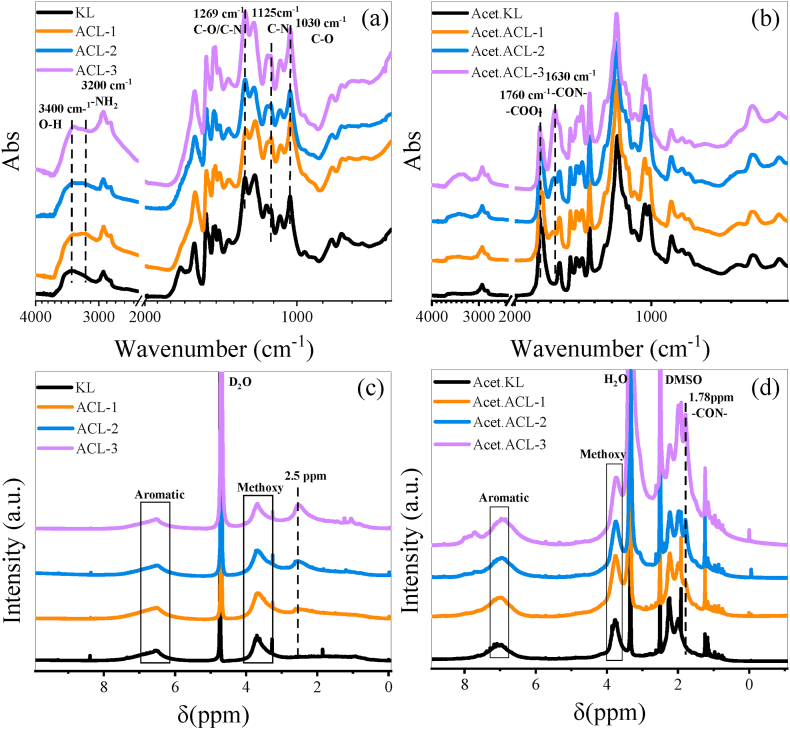
(a, c) FT-IR and ^1^H NMR spectra of KL and ACL (b, d) FT-IR and ^1^H NMR spectra of Acet.KL and Acet.ACL.

The FT-IR spectra of KL and ACL are shown in Fig. 1(a). The characteristic absorption peaks of lignin are observed in all spectra. The intensities of the N−H absorption peak at 3200 cm^−1^ and C−N absorption peak at 1125 cm^−1^ are higher in ACL than those in KL, indicating the introduction of amine groups to KL [27,28]. Furthermore, the peak at 1269 cm^−1^ corresponds to C−O and C−N stretching, whereas that at 1030 cm^−1^ corresponds to C−O stretching [29,30]. The KL and ACL samples were acetylated to confirm these findings. The FT-IR spectra of acetylated KL (Acet.KL) and acetylated ACL (Acet.ACL) are shown in Fig. 1(b). Compared with those of Acet.KL, the spectra of the Acet.ACL samples exhibit a distinct absorption peak of −CON− (amide) at 1646 cm^−1^ and of −COO− (ester) stretching at 1760 cm^−1^ [31].

The ^1^H NMR spectra of KL and ACL samples are shown in Fig. 1(c). Peaks are observed at approximately δ 7 and 3.5 ppm, corresponding to aromatic and methoxy protons [32]. Compared with KL, the peak at δ 2.5 ppm in ACL indicates the presence of the −CH_2_−N− moiety of CEH, and the intensity of the peak becomes more pronounced with increasing CEH content [33]. The results obtained after the acetylation are shown in Fig. 1(d). When comparing the spectra of the Acet.KL and Acet.ACL samples, the peak at δ 1.78 ppm becomes more distinct with the increasing amount of CEH, confirming successful amination [17].

**Fig. 2:**
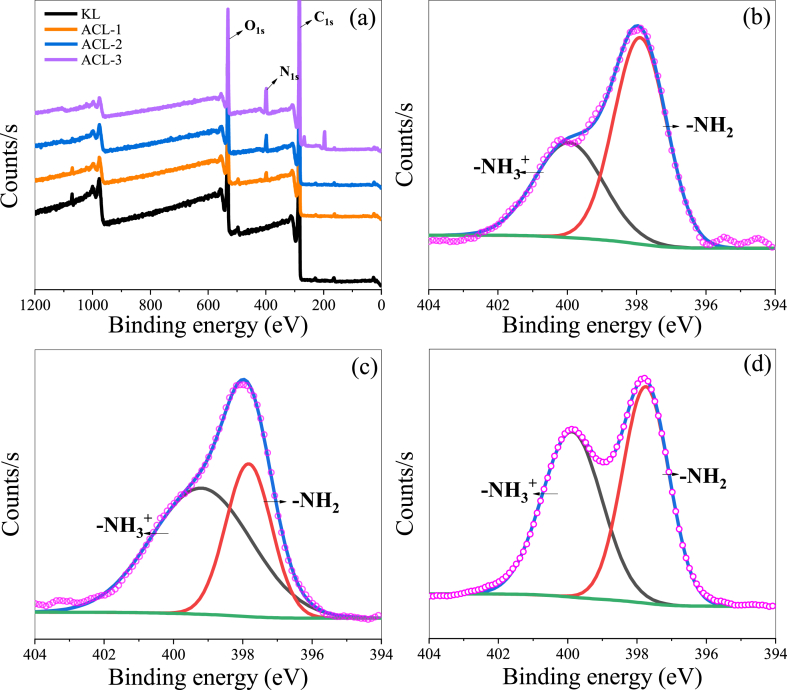
(a) XPS survey scan spectra of KL and ACL, (b) N1s core-level spectrum of ACL-1, (c) N1s core-level spectrum of ACL-2, and (d) N1s core-level spectrum of ACL-3.

Fig. 2(a) shows the XPS spectra of the KL and ACL samples. XPS is a surface-sensitive technique used to analyze the elemental composition, chemical state, and electronic state of materials. This technique is based on the principle that X-rays excite core-level electrons from atoms near the material surface. Information regarding the elements present in the sample and their chemical environments can be inferred by measuring the kinetic energy of the emitted electrons. In the XPS spectrum of the KL sample, peaks corresponding to the C1s (carbon) and O1s (oxygen) core levels are detected at approximately 283 and 530 eV, respectively, which indicates the presence of carbon and oxygen in lignin. After amination, the spectrum of the ACL sample exhibits an additional N1s (nitrogen) peak at 398 eV, which is not present in that of the KL sample. As the CEH content increases, the intensity of this peak increases, indicating that the nitrogen content increases proportionally with the CEH content. The N1s core-level spectrum of ACL can be deconvoluted into two distinct peaks at approximately 397.7 and 400.0 eV, which are attributed to the amino (–NR_2_) and ammonium (–NH_3_^+^) groups, respectively [34]. These results confirm that N1s exist in two different forms in the ACL sample. The higher intensity of the N1s peak in the ACL sample than that in the KL sample indicates the successful introduction of amine groups.

#### 3.1.3 Thermal properties of ACL

**Fig. 3:**
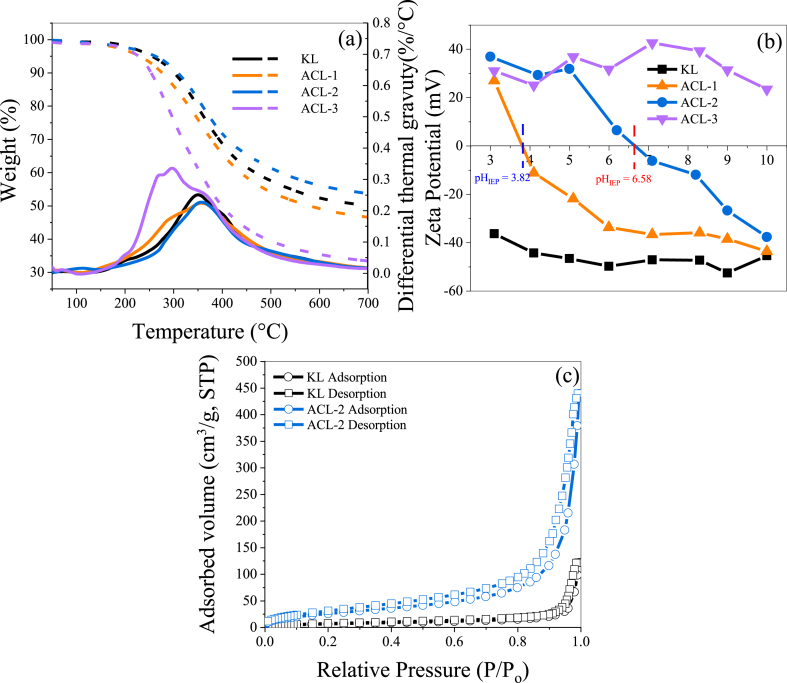
(a) TGA and DTG curves, (b) zeta potential, and (c) N_2_ adsorption–desorption isotherms of KL and ACL.

The thermal characteristics of the lignin samples were determined using TGA. Fig. 3(a) displays the TGA and derivative thermogravimetry (DTG) curves of KL and ACL in the temperature range of 50–700 °C. The thermal decomposition of lignin occurs gradually over a wide temperature range up to 700 °C. This slow decomposition is primarily attributed to the complex structure of lignin, particularly the presence of various aromatic rings and functional groups [35]. The maximum decomposition temperatures of KL, ACL-1, ACL-2, and ACL-3 are 351, 359, 359, and 294 °C, respectively. ACL-1 and ACL-2 exhibit higher maximum decomposition temperatures than those of KL, which can be ascribed to the enhanced bonding through amination, resulting in condensation or crosslinking reactions within the lignin macromolecules. Conversely, ACL-3 shows a lower maximum decomposition temperature than that of KL, which can be attributed to its higher nitrogen content (6.1 %), indicating potential reactions between the amino groups, resulting in weak bonds within the molecule, thus lowering the thermal stability. The residual mass percentages of KL, ACL-1, ACL-2, and ACL-3 above 700 °C are 50 %, 47 %, 54 %, and 33 %, respectively. As expected, ACL-3 exhibits a lower residual mass than that of KL because of the formation of weak bonds via amino group reactions. However, compared to KL, ACL-2 exhibits a higher residual mass, whereas ACL-1 exhibits a lower residual mass, which can be attributed to the high polydispersity index of ACL-1 (4.61), indicating its non-uniform chemical structure and low thermal stability. Therefore, among the three compounds, only ACL-2 achieved higher thermal stability than KL, suggesting the strong intermolecular interactions induced in ACL-2.

#### 3.1.4 Surface charge properties of ACL

The influence of the amine content on the surface charge of ACL was evaluated through zeta potential analysis in the pH range of 3–10. As illustrated in Fig. 3(b), KL consistently exhibits a high negative charge across the entire pH range because of its abundant hydroxyl groups, which remained negatively charged throughout the pH spectrum. For ACL-1 and ACL-2, the introduction of amine functionalities results in the transition to negative charges at pH 3.82 and 6.58, respectively. This transition point can be attributed to the typical behavior of amine groups, which tend to accept hydrogen ions to become positively charged under acidic conditions and release hydrogen ions to become negatively charged under alkaline conditions. However, ACL-3 consistently exhibits a highly positive charge over the entire pH range because of its exceptionally high amine content; in other words, the amine groups continuously accept hydrogen ions. Additionally, the significant cross-linking in ACL-3 causes a substantial size increase, whereby more amine groups are incorporated into the crosslinked structure, which continues to sustain a positive charge. Ultimately, the variation in the zeta potential with pH is considered an important parameter that can greatly affect the adsorption of both cationic and anionic dyes [36]. When the zeta potential is positive, electrostatic repulsion with cationic dyes may occur, thereby reducing the adsorption efficiency. Therefore, ACL-3 was deemed unsuitable for cationic-dye adsorption because of its positive charge, which hindered successful adsorption. Conversely, ACL-1 and ACL-2 were demonstrated to adsorb both cationic and anionic dyes as their charges switched between positive and negative depending on the pH.

#### 3.1.5 Specific surface areas and pore sizes of ACL

Table 2 summarizes the surface areas, pore volumes, and pore sizes of the lignin samples. The surface area of KL is 24.40 m^2^·g^−1^, whereas the surface area of ACL-2 significantly increases to 101.21 m^2^·g^−1^, depicting a tenfold augmentation. A larger surface area implies that ACL provides a more substantial adsorption surface, demonstrating superior dye adsorption capabilities. Furthermore, the variations in the pore volume and size align with the changes in the surface area. All samples exhibit type-IV N_2_ adsorption–desorption isotherms, according to the International Union of Pure and Applied Chemistry, indicating a mesoporous structure with a mean pore size range of 2–50 nm [37]. Pore size analysis using the BJH method confirms mesoporous characteristics in the pore size range of 17–30 nm for all samples [37]. The pore volumes for KL and ACL-2 are 0.18 and 0.68 cm^3^·g^−1^, respectively.

**Table 2:** Surface area, pore volume, and pore size of KL and ACL-2.

Entry	Specific surface area (m^2^·g^−1^)		Pore volume (cm^3^·g^−1^ × 10^3^)	Pore size (nm)
	S_BET_	S_BJH_	V_BJH_	D_BJH_
KL	24.40	16.68	0.18	3.82
ACL-2	101.21	125.16	0.68	24.66

### 3.2 Effect of operational parameters on dye adsorption

#### 3.2.1 Effect of solution pH, adsorption time, and temperature on dye removal

**Fig. 4:**
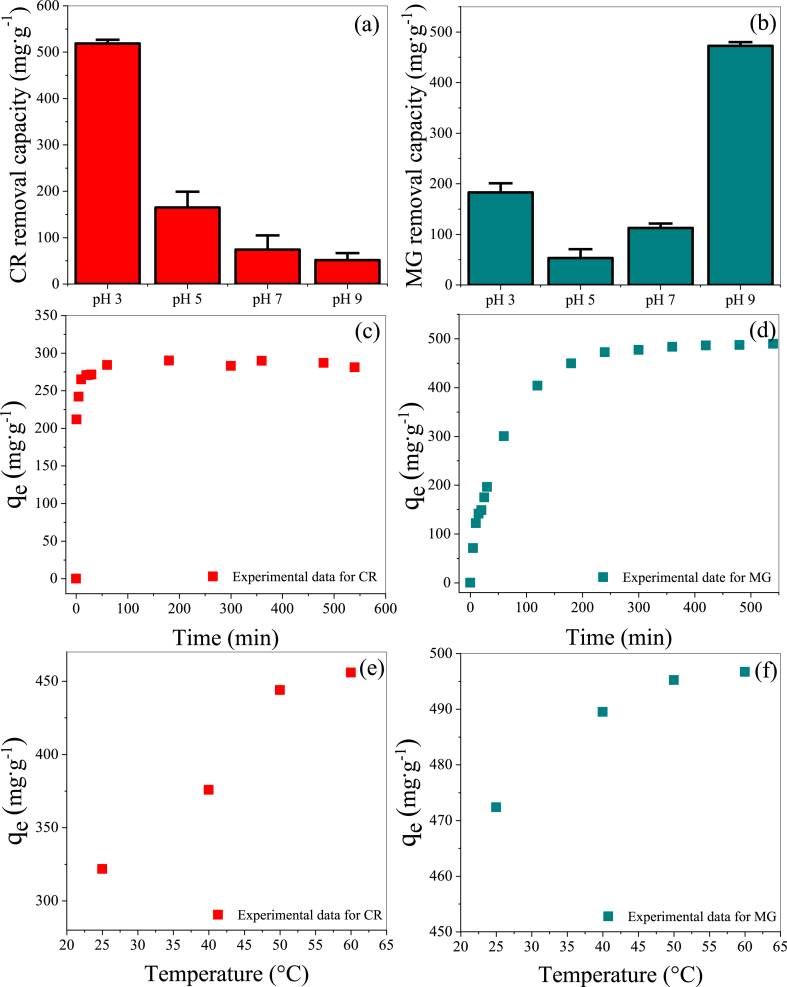
Effect of (a, b) pH (CR concentration: 700 mg·L^−1^, MG concentration: 400 mg·L^−1^, adsorbent mass: 0.03 g, temperature: 25 °C, contact time: 6 h), (c, d) time (CR concentration: 700 mg·L^−1^, CR pH: 3, MG concentration: 400 mg·L^−1^, MG pH: 9, adsorbent mass: 0.03 g, temperature: 25 °C), and (e, f) temperature (CR concentration: 700 mg·L^−1^, MG concentration: 400 mg·L^−1^, adsorbent mass: 0.03 g, contact time: 6 h) on the CR and MG adsorption capacities of ACL-2.

pH is a crucial parameter in the adsorption behavior, exerting a significant influence on the interactions between the dyes and adsorbents [38,39]. The adsorption capacities of CR and MG as a function of the pH values are shown in Fig. 4(a) and (b). For the negatively charged CR, the adsorption capacity increases with decreasing pH, reaching 518.59 mg·g^−1^ at pH 3. By contrast, the positively charged MG exhibits the highest adsorption capacity (472.57 mg·g^−1^) at the alkaline condition of pH 9. This behavior can be explained by investigating the zeta potential of ACL-2. As ACL-2 displayed a positive charge at a low pH value, which gradually decreased and shifted to negative values as the pH value increased, the electrostatic attraction of ACL-2 with the anionic dye CR increased, and that with the cationic dye, MG decreased under acidic conditions, resulting in the observed adsorption capacity. Conversely, under alkaline conditions, the electrostatic attraction with MG increased, and that with CR decreased, resulting in a higher adsorption capacity for MG. This adsorption mechanism also involves hydrogen bonding, NH−π, and π−π interactions [40,41]. Therefore, the selective adsorption of both anionic and cationic dyes was achieved by adjusting the pH alone, demonstrating the versatility of the adsorption process.

Fig. 4(c) and (d) show the effect of adsorption time on the CR and MG adsorption capacities of ACL-2. For CR, the amount removed increases rapidly from 0 to 90 min and remains stable thereafter. For MG, the amount removed increases rapidly from 0 to 180 min and then reaches equilibrium. Although CR reaches the adsorption equilibrium faster than MG, the removal efficiency of MG is higher than that of CR when equilibrium is attained.

Fig. 4(e) and (f) show the CR and MG adsorption capacities of ACL-2 at various temperatures (25, 40, and 60 °C). The impact of temperature on CR and MG adsorption reveals that the CR and MG adsorption capacities of ACL-2 increase with increasing temperature. These findings suggest that higher temperatures may accelerate the material transfer rates during the adsorption process or enhance chemical interactions [42]. Thus, the temperature-dependent results underscore the importance of temperature as a critical factor for enhancing the adsorption characteristics of the material [43].

### 3.3 Analysis of CR and MG adsorption kinetics and isotherms

#### 3.3.1 CR and MG adsorption kinetics

Adsorption kinetics were investigated to determine the impact of adsorption time on the adsorption capacity to elucidate the adsorption mechanisms of CR and MG [44]. The commonly used pseudo-first- and second-order models were employed to analyze the adsorption process. The nonlinear pseudo-first- and second-order kinetic equations are given by Equations (3), (4), respectively [45,46]:

(3) $$q_{e\;}=q_{e}\left(1-e^{-k_{1}t}\right)\text{,}$$

(4) $$q_{t}=\frac{q_{e}^{2}k_{2}t}{1+q_{e}k_{2}t}\text{,}$$

where *q*_*e*_ is the equilibrium adsorption capacity (mg·g^−1^); *q*_*t*_ is the adsorption capacity (mg·g^−1^) at time *t* (min); and *k*_*1*_ and *k*_*2*_ are the kinetic rate constants for pseudo-first and second-order kinetic models, respectively.

**Fig. 5:**
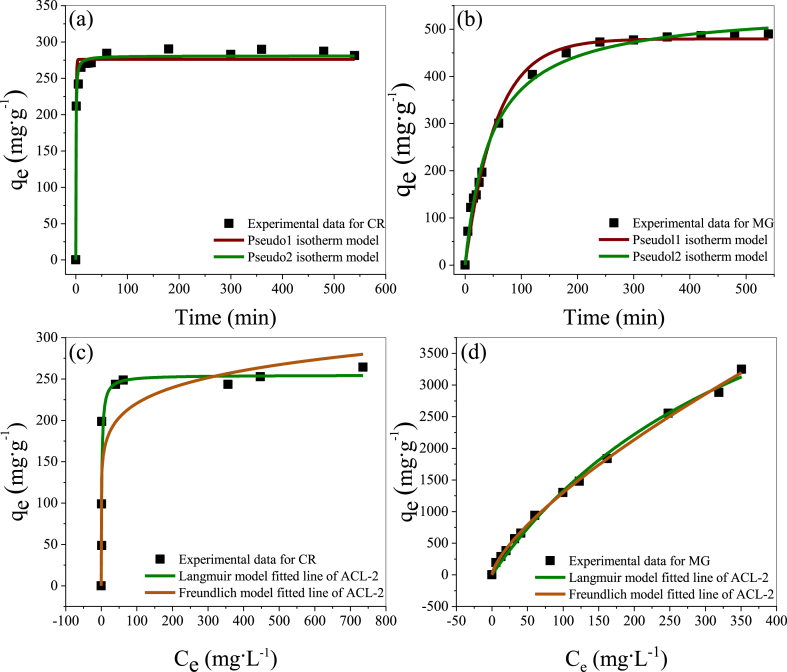
Nonlinear fitting curves of the (a) pseudo-first-order and (b) pseudo-second-order kinetic models (CR concentration: 700 mg·L^−1^, CR pH: 3, MG concentration: 400 mg·L^−1^, MG pH: 9, adsorbent mass: 0.03 g, temperature: 25 °C). Langmuir and Freundlich models (CR pH: 3, MG pH: 9, adsorbent mass: 0.03 g, temperature: 25 °C, contact time: 6 h) for the (c) CR and (d) MG adsorption onto ACL-2.

The pseudo-first- and second-order models are suitable for describing physical and chemical adsorptions, respectively [47]. The fitting curves for the pseudo-first and second-order models for CR and MG are shown in Fig. 5(a) and (b), respectively, and the parameters of the kinetic equations are listed in Table 3. For both CR and MG, the correlation coefficients (R^2^) for the pseudo-second-order model are higher, approaching 1, than those of the pseudo-first-order model. Therefore, the fitting results for the time-dependent dye adsorption by ACL-2 suggest that the pseudo-second-order model is more suitable than the pseudo-first-order model. This observation supports the assumption that the adsorption of CR and MG by ACL-2 is primarily driven by chemical adsorption and electrostatic interactions. The observed increase in the chemical adsorption with temperature corresponds well with the theoretical expectations, thereby further validating the results.

**Table 3:** Characteristic parameters of the kinetic equation for the adsorption of CR and MG onto ACL-2.

Isotherms	Parameters	CR	MG
Pseudo-first-order kinetics	R^2^	0.973	0.990
	k_1_ (min^−1^)	1.449	0.018
	q_e(cal)_ (mg·g^−1^)	275.912	479.386
	SD (mg·g^−1^)	4.06	2.809
Pseudo-second-order kinetics	R^2^	0.989	0.994
	k_2_ (g·mg^−1^ min^−1^)	0.009	3.918 × 10^−5^
	q_e(cal)_ (mg·g^−1^)	280.770	545.949
	SD (mg·g^−1^)	7.114	8.524

SD* denotes “standard deviation.”

#### 3.3.2 CR and MG adsorption isotherms

Adsorption isotherms were used to analyze the influence of the initial adsorbate concentration on the adsorption capacity [45]. Fig. 5(c) and (d) illustrate the effect of the initial adsorbate concentration on the adsorption capacity of ACL-2. The adsorption capacity of CR initially increases up to the equilibrium value, whereas that of MG continuously increases. The Langmuir and Freundlich adsorption isotherm models were employed to apply the adsorption isotherms. The nonlinear Langmuir and Freundlich equations are expressed as Equations (5), (6), respectively [14,48]:

(5) $$q_{e}=\frac{q_{m}k_{L}C_{e}}{1+k_{L}C_{e}}\text{,}$$

(6) $$q_{e}=k_{F}C_{e}^{\frac{1}{n}}\text{,}$$

where *q*_*e*_ is the equilibrium adsorption capacity (mg·g^−1^), *C*_*e*_ is the concentration of the adsorbate at equilibrium (mg·L^−1^), *q*_*m*_ is the maximum adsorption capacity (mg·g^−1^), *k*_*L*_ is the Langmuir constant, *k*_*F*_ is a constant representing the multilayer adsorption capacity, and *n* is an empirical parameter related to the intensity of adsorption.

The Langmuir and Freundlich models assume monolayer and heterogeneous multilayer adsorptions, respectively [49,50]. Fig. 5(c) and (d) show the experimental data fitting of the Langmuir and Freundlich adsorption isotherm models. The relevant nonlinear equation parameters are listed in Table 4. For CR, the R^2^ value for the Langmuir model is higher than that for the Freundlich model, approaching 1, indicating a better fit. Conversely, for MG, the R^2^ value of the Freundlich model is higher than that of the Langmuir model, approaching 1. Accordingly, CR can be assumed to be more suitable for the Langmuir model, suggesting monolayer adsorption, whereas MG is more suitable for the Freundlich model, implying multilayer adsorption.

**Table 4:** Characteristic parameters of the Langmuir and Freundlich models for the adsorption of CR and MG onto ACL-2.

Isotherms	Parameters	CR	MG
Langmuir isotherm	R^2^	0.864	0.995
	k_L_ (L·mg ^−1^)	0.594	0.002
	q_m(cal)_ (mg·g^−1^)	245.559	6862.605
	SD (mg·g^−1^)	18.648	592.923
Freundlich isotherm	R^2^	0.798	0.998
	k_F_ (mg·g^−1^)	127.261	47.38
	n	0.12	0.72

SD* denotes “standard deviation”.

#### 3.3.3 CR and MG adsorption thermodynamic studies

The thermodynamic parameters, including changes in standard free energy (ΔG°), enthalpy (ΔH°), and entropy (ΔS°), can be estimated from the adsorption isotherm data measured at various temperatures using Equations (7), (8), (9), respectively [51]:

(7) $$K_{d}=\lim_{C_{e}\to 0}q_{e}/C_{e}\text{,}$$

(8) $$\Delta G{}^{\circ}=-\text{RT}\;\ln\;K_{d}\text{,}$$

(9) $$\ln\;K_{d}=\frac{\Delta S{}^{\circ}}{R}-\;\frac{\Delta H{}^{\circ}}{R}\frac{1}{T}\text{,}$$

where *K*_*d*_ is the adsorption distribution coefficient obtained by plotting ln (q_e_·C_e_^−1^) versus Ce at different temperatures and extrapolating to zero Ce, ΔH° is the standard enthalpy change (kJ·mol^−1^), ΔS° is the standard entropy change (J·mol^−1^ K^−1^), ΔG° is the standard free energy change (kJ·mol^−1^), *R* is the gas constant (8.314 J mol^−1^ K^−1^), and *T* is the absolute temperature (K).

The thermodynamic parameters are listed in Table S2, and the relationship between ln (*K*_*d*_) and 1/T is shown in Fig. S2. CR and MG exhibit decreasing trends in adsorption capacity with increasing temperature, indicating the exothermic nature of dye adsorption [52].

### 3.4 Reusability

**Fig. 6:**
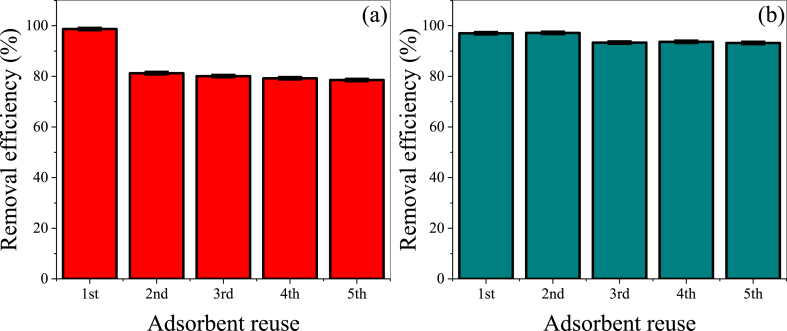
Reusability test results for the (a) CR and (b) MG adsorption onto ACL-2 (CR concentration: 700 mg·L^−1^, CR pH: 3, MG concentration: 400 mg·L^−1^, MG pH: 9, adsorbent mass: 0.03 g, contact time: 4 h, temperature: 25 °C).

Effective desorption and sustained reusability are critical factors for evaluating the adsorbent performance [53,54]. The reusability of the adsorbent is crucial, given the persistent nature of water pollution. Therefore, we investigated the potential of reusing the ACL-2 sample by desorbing the dyes from the adsorbent using DW/ethanol (5:5) after removing CR and MG. The CR and MG adsorption efficiencies of ACL-2 over five cycles are shown in Fig. 6. For CR, the adsorption efficiency sharply decreases by approximately 18 % from the second cycle onward, displaying a gradual decline with an increasing number of cycles. By contrast, for MG, the adsorption efficiency remains relatively stable, showing a decrease of approximately 4 % from the third cycle onward while maintaining equilibrium. Thus, even after five cycles, the CR adsorption efficiency remains above 78 %, and that of MG consistently exceeds 93 %.

The results of the reusability test align with the discussed mechanism, in which the characteristics of CR are closer to those of the Langmuir model (monolayer adsorption). By contrast, those of MG are similar to that of the Freundlich model (multilayer adsorption). Multilayer adsorption is advantageous for desorption and re-adsorption as it allows the effective utilization of the spaces between the adsorption layers [55]. Consistent with this mechanism, MG exhibits a lower loss of adsorption efficiency than CR during reuse.

As shown in Fig. S5, the FT-IR measurements of lignin after desorption and drying indicate minimal structural deformation, with the FT-IR spectrum closely resembling that of lignin before adsorption. This suggests that lignin retains its original properties and can be reused multiple times in the dye removal process, highlighting its potential as an economical and environmentally friendly method. Ultimately, ACL-2 enables effective desorption and provides stable and reversible adsorption efficiency even upon reuse.

### 3.5 Possible mechanism for MB and CR adsorption

Fig. S3 shows the FT-IR spectra of ACL-2 before and after CR and MG adsorption. The intensity of the peaks at 1587 and 1364 cm^−1^, corresponding to N=N and C–N bonds, respectively, increases in the CR spectra [56,57]. Additionally, new peaks at 1159, 1033, and 757 cm^−1^, attributed to R–SO3–, S=O, and C–S, respectively, emerge after CR adsorption [[58], [59], [60]]. These peaks closely resemble the FT-IR peaks of CR. For MG, a similar increase in the peak intensity is observed at 1587 and 1364 cm^−1^ [56,57]. These results confirm the successful adsorption of CR and MG onto ACL-2.

Fig. S4 shows the surface morphology and elemental composition of ACL-2 before and after CR and MG adsorption. The surface elemental compositions of ACL-2 before and after adsorption were analyzed using SEM-EDS, revealing the elevated levels of nitrogen and sulfur after CR adsorption. For MG adsorption, ACL-2 exhibits increased nitrogen, sulfur, and chlorine contents compared to after adsorption. This increase implies the effective adsorption of the dyes onto the ACL-2 surface.

After amination, the specific surface area remarkably varies. In particular, the specific surface area of the unmodified KL is 24.40 m^2^·g^−1^, whereas that of ACL-2 is 101.21 m^2^·g^−1^, which is approximately 4.1 times higher. Therefore, ACL-2 provides greater accessibility to the active sites of CR and MG because of its higher specific surface area, thereby facilitating a more efficient adsorption [61]. Generally, a higher specific surface area provides a larger contact area between the adsorbent and adsorbate, increasing the availability of adsorption sites for interaction. Consequently, ACL with a larger specific surface area offers improved adsorption performance for CR and MG, highlighting its potential as an effective adsorbent for wastewater treatment.

According to the adsorption kinetics, both CR and MG exhibit behaviors that closely fit pseudo-second-order kinetic models, suggesting chemical adsorption [62]. Considering the pH-dependent electrostatic interactions, these interactions contribute significantly to the adsorption mechanism. The Langmuir model is suitable for describing CR adsorption, whereas the Freundlich model fits well for MG adsorption. These findings indicate that CR tends toward monolayer adsorption, whereas MG tends toward multilayer adsorption [63]. These results are consistent with the findings of the reusability tests. Furthermore, the analysis of adsorption as a function of temperature reveals the exothermic reactions for both CR and MG adsorption [64].

**Fig. 7:**
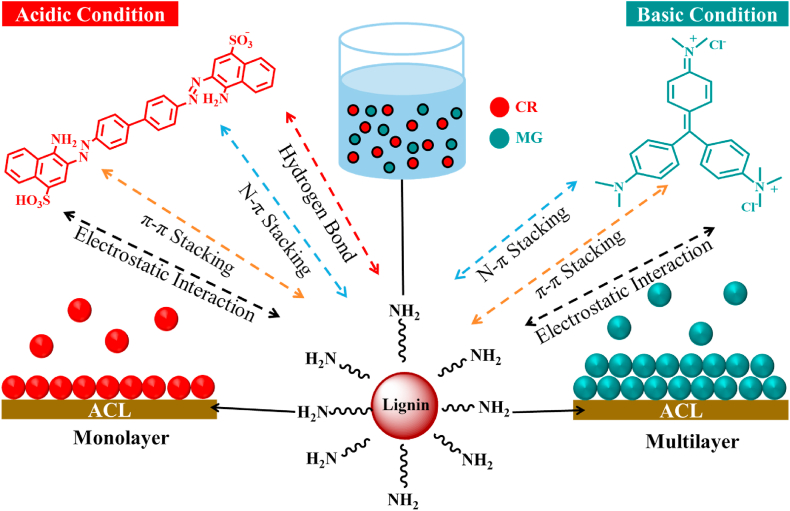
Possible adsorption mechanism of CR and MG on ACL.

For the interactions between CR and MG, the adsorption mechanisms include various forces and bonds that enhance the adsorption capacity. CR adsorption is primarily facilitated by hydrogen bonding, NH–π interactions, π–π stacking interactions, and electrostatic interactions [65]. Hydrogen bonding occurs between the hydroxyl or amino groups on the lignin surface and azo groups of CR, whereas NH–π and π–π interactions involve the aromatic rings of CR and π-electrons from the lignin structure. Notable electrostatic interactions are noted under acidic conditions because of the positive charge of the protonated amino groups on the lignin and the negative charge of anionic CR. The adsorption mechanisms of MG include NH–π, π–π stacking, and electrostatic interactions [66]. Under alkaline conditions, the lignin particles acquire a negative charge, which enhances their electrostatic attraction with the cationic MG dye. NH–π and π–π stacking interactions also play critical roles, where the aromatic rings and π-electrons of MG interact with the lignin surface. These interactions collectively contribute to the high adsorption capacities of CR and MG. Furthermore, NH–π and π–π adsorption mechanisms are observed, as shown in Fig. 7, indicating that the adsorbent can selectively interact with dyes based on these interactions depending on the pH value. These results highlight the remarkable adsorption capacity of this adsorbent compared with that of previously reported biomass-based adsorbents.

## 4 Conclusions

ACL was successfully synthesized via amination and exhibited an enhanced molecular weight and thermal stability. Additionally, its specific surface area was considerably larger than that of KL, confirming its potential as an effective adsorbent. ACL exhibited highly efficient adsorption capacity for both anionic and cationic dyes under various pH conditions. The kinetics models for CR and MG fitted well with the pseudo-second-order model with the maximum adsorption capacities of 280.77 and 545.95 mg g^−1^, respectively. The Langmuir model was suitable for the CR dye, whereas the Freundlich model was suitable for the MG dye. Based on the reusability tests, ACL-2 demonstrated effective desorption and sustained reusability for both CR and MG, with MG exhibiting a more stable adsorption efficiency over multiple cycles owing to its behavior resembling the Freundlich model. The presumed adsorption mechanisms for CR and MG primarily involves electrostatic interactions. Therefore, ACL-2 synthesized in this study could selectively remove anionic and cationic dyes through pH control. In addition, it provides a competitive advantage with higher adsorption capacities than those reported in previous studies, demonstrating its ability as a low-cost and high-performance adsorbent.

## Data availability statement

Data included in article/supplementary material/referenced in article.

## CRediT authorship contribution statement

**Do Hun Oh:** Writing – review & editing, Writing – original draft, Methodology, Investigation, Formal analysis, Conceptualization. **Ji Won Heo:** Writing – review & editing, Investigation, Conceptualization. **Qian Xia:** Writing – review & editing, Investigation. **Min Soo Kim:** Writing – review & editing, Conceptualization. **Yong Sik Kim:** Writing – original draft, Supervision, Methodology, Funding acquisition, Conceptualization.

## Declaration of competing interest

The authors declare that they have no known competing financial interests or personal relationships that could have appeared to influence the work reported in this paper.
