# Supplementary material for: Amine-crosslinked lignin for water pollution attributable to organic dye remediation: Versatile adsorbent for selective dye removal and reusability
Source: Heliyon. 2024 Sep 5;10(17):e37497. doi: 10.1016/j.heliyon.2024.e37497 (PMC11407063; doi:10.1016/j.heliyon.2024.e37497)
Supplement: Multimedia component 1 [file mmc1.docx]

**Supplementary data:**

**Amine-crosslinked lignin for water pollution attributable to organic dye remediation: Versatile adsorbent for selective dye removal and reusability**

Do Hun Oh, Ji Won Heo, Qian Xia, Min Soo Kim, and Yong Sik Kim^*^

Department of Paper Science & Engineering, College of Forest and Environmental Sciences, Kangwon National University, Chuncheon 24341, Republic of Korea

Fig. S1. FT-IR of spectra of (a) KL and ACL, and (b) Acet.KL and Acet.ACL.

Table S1. Elemental contents of KL and ACL

| Entry | Elemental content | | |
| --- | --- | --- | --- |
|  | C (%) | N (%) | S (%) |
| KL | 66.32 | 0.4 | 1.89 |
| ACL (2h) | 64.77 | 3.2 | 1.59 |
| ACL (4h) | 64.59 | 3.0 | 1.61 |
| ACL (6h) | 63.51 | 3.3 | 0.80 |
| ACL (12h) | 64.51 | 3.3 | 0.88 |
| ACL (24h) | 63.10 | 3.6 | 0.76 |

Fig. S2. Plot of ln (K_d_) of ACL-2 versus inverse temperature (1/T); (a) for the CR adsorption; (b) for MG adsorption

Table S2. Characteristic parameters of thermodynamic studies for the adsorption of CR and MG onto ACL-2

| Adsorbates | Samples | Parameters | | | | |
| --- | --- | --- | --- | --- | --- | --- |
|  |  | Temperature (K) | K_d_ | ∆G° (kJ·mol^−1^) | ∆H°  (kJ·mol^−1^) | ∆S°  (J·mol^−1^·K^−1^) |
| CR | AL-2 | 298.15 | 0.85 | 0.40 | 18.61 | 60.92 |
|  |  | 313.15 | 1.159 | -0.38 |  |  |
|  |  | 323.15 | 1.73 | -1.48 |  |  |
|  |  | 333.15 | 1.87 | -1.73 |  |  |
| MG | AL-2 | 298.15 | 17.09 | 7.04 | 51.17 | 195.26 |
|  |  | 313.15 | 46.65 | 10.00 |  |  |
|  |  | 323.15 | 104.33 | 12.49 |  |  |
|  |  | 333.15 | 149.58 | 13.87 |  |  |

Fig. S3. FT-IR spectra after adsorption of CR and MG on ACL-2


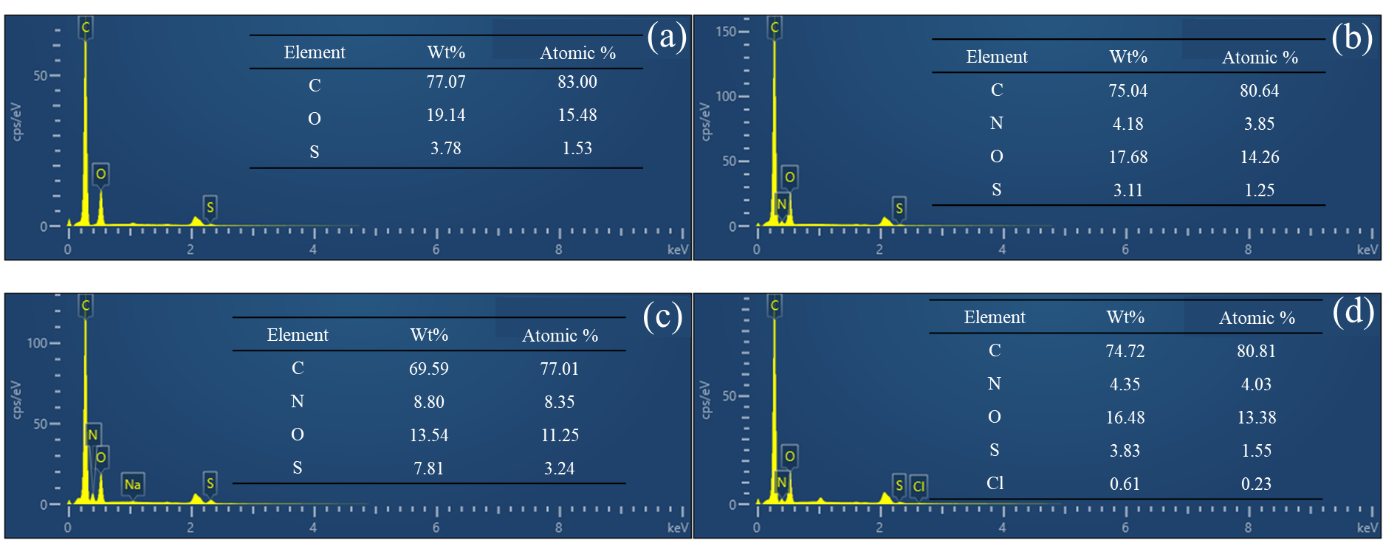


Fig. S4. (a) Surface element content of KL before adsorption, (b) Surface element content of ACL-2 before adsorption, (c) Surface element content of ACL-2 after adsorption of CR, and (d) Surface element content of ACL-2 after adsorption of MG

**S1. Reusability**

FT-IR measurements were conducted after desorption and drying to determine whether deformation occurred in lignin during the desorption and drying process following dye adsorption. As depicted in Fig. S5, the FT-IR spectrum after desorption and drying closely resembles the spectrum of ACL-2 before adsorption, compared to the spectrum after dye adsorption. This observation indicates minimal structural modification of lignin during the desorption and drying process. In other words, lignin retains most of its original structural properties even after dye adsorption, suggesting that it can be reused following desorption and drying. Furthermore, a comparison of the main peaks of the FT-IR spectra revealed that nearly identical peaks appeared in lignin before and after adsorption. This confirms that the main functional groups of lignin were not damaged. It was observed that the primary peaks associated with lignin remained predominant rather than those related to the dye. These findings imply that lignin can be reused multiple times in the dye removal process, supporting its potential as an economical and environmentally friendly dye removal method. This property could be a significant advantage in industrial applications and underscores the need for further research to enhance the reusability of lignin.

Fig. S5. (a) FT-IR spectra of ACL-2 before CR adsorption, after CR adsorption, and after CR desorption, (b) FT-IR spectra of ACL-2 before MG adsorption, after MG adsorption, and after MG desorption
